# Trends of *Mycobacterium tuberculosis* and rifampicin resistance in Adigrat General Hospital, Eastern zone of Tigrai, North Ethiopia

**DOI:** 10.1186/s40794-020-00115-1

**Published:** 2020-08-28

**Authors:** Getachew Kahsu Abay, Bahlbi Hailay Abraha

**Affiliations:** 1 Department of Medical Laboratory, College of Medicine and Health Sciences, Adigrat University, P. O. Box 50, Adigrat, Ethiopia; 2 Department of Medical Laboratory, Adigrat General Hospital, Adigrat, Ethiopia

**Keywords:** Adigrat general hospital, Rifampicin-resistant, *Mycobacterium tuberculosis*, Tigrai, Ethiopia

## Abstract

**Background:**

*Tuberculosis* is an infectious disease usually caused by *Mycobacterium tuberculosis* bacteria. The global emergence of mono- or multidrug-resistant tuberculosis and extensively drug-resistant forms of tuberculosis pose a considerable challenge to tuberculosis control programs. There has been no reliable and organized data on trends and drug resistance of *Mycobacterium tuberculosis* in the study area. Therefore, this study aimed to determine the trends of *Mycobacterium tuberculosis* and rifampicin resistance in the Adigrat General Hospital, eastern Zone of Tigrai, North Ethiopia.

**Methods:**

A hospital-based retrospective cross-sectional study was conducted at Adigrat General Hospital from January 2015 to 2018.Data was collected retrospectively from the GeneXpert*™* TB registration book using a data extraction format. Data was entered into Epi-Info 3.1 and subsequently exported and analyzed using SPSS Version 20.The results were summarized using descriptive statistics, tables, and figures. Bivariate and multi-variant regression analysis was employed to measure the association between dependent and independent variables. *P* values < 0.05 were considered statistically significant.

**Result:**

A total of 5944 *Mycobacterium tuberculosis* presumptive patients were included in the study. The majority of the study participants were male (58.1%) with participants’ median age of 40.0 (IQR 26–57) years, the majority were 30–44 years. The overall positive cases of *Mycobacterium tuberculosis* was 24.3% (1446) with a total of 132 (9.1%) found to be resistant to rifampicin. Of the total confirmed positive cases 8.7% (103/1188) and 11.2% (29/258) were rifampicin resistance of presumptive tuberculosis and presumptive drug resistance tuberculosis patients respectively. Age, the reason for diagnosis, site of presumptive tuberculosis, and/or being HIV infected showed significant association with our dependent variable; however, only age and being HIV infected were associated with rifampicin resistance.

**Conclusion:**

In our study, the overall trends of *Mycobacterium tuberculosis* and rifampicin resistance were found to be high. Rifampicin resistance is more common in patients with HIV and presumptive drug resistance tuberculosis individuals. Therefore, maximizing early detection of drug-resistant and strengthening tuberculosis infection control activities are recommended to reduce the burden of this contagious and potentially deadly disease.

## Introduction

Tuberculosis (TB) is caused by a bacterium called *Mycobacterium tuberculosis*. The bacteria usually attack the lungs, but TB bacteria can attack any part of the body, such as the kidney, spine, and brain. Not everyone infected with TB bacteria becomes sick. As a result, two TB-related conditions exist: latent TB infection (LTBI) and TB disease. If not treated properly, TB disease can be fatal. Tuberculosis is spread from person to person through the air. When people with lung TB cough, sneeze or spit, they propel the TB germs into the air. A person needs to inhale only a few of these germs to become infected. Tuberculosis is curable and preventable [1, 2]. A relatively small proportion of people infected with *Mycobacterium tuberculosis* will go on to develop TB disease; however, the probability of developing TB is much higher among people with immune suppression or compromise. About one-quarter of the world’s population has latent TB, which means people have been infected with TB bacteria but are not yet ill with the disease and cannot transmit the disease [2].

Tuberculosis (TB) has existed for millennia and remains a major global health problem. It causes ill-health of approximately 10 million people each year and is one of the top ten causes of death worldwide [3]. According to the Global tuberculosis Report (2017), 10.4 million people have estimated the incidence to have all forms of TB in 2016 while an estimated 1.3 million people died from TB, excluding deaths attributed to TB/HIV in combination. In addition, an estimated 4.1% of these new TB cases and 19% of the previously treated cases are believed to harbor drug resistant-TB with an estimated 240,000 deaths annually due to multi-drug resistant tuberculosis (MDR-TB) [3]. Human immunodeficiency virus (HIV) is an infection that attacks the body’s immune system, specifically the white blood cells called CD4 cells. HIV destroys these CD4 cells, weakening a person’s immunity against infections such as tuberculosis and some cancers. The risk of developing tuberculosis (TB) is estimated to be between 16 and 27 times greater in people living with HIV than among those without HIV. The World Health Organization (WHO) estimates that 4.5 million people are co-infected with HIV and TB globally [1, 2, 4].

Ethiopia is among the 30 highest TB, HIV, and MDR-TB burden countries, which accounted for 80% of all estimated TB cases worldwide. Ethiopia had an annual estimated TB incidence of 207/100,000 populations and a death rate of 33 per 100,000 populations in 2014 [5]. Among the notified TB cases in 2014, 1300 (1.6%) of new TB cases and 11.8% of previously treated TB cases were drug-resistant [3]. Besides, a drug resistance (DR-TB) sentinel report in 2013 showed the MDR-TB prevalence of 2.3% among new and 17.8% among previously retreated TB cases in Ethiopia [6] .In the same year, there was notification of 119, 592 new TB cases and enrollment of 597 DR-TB cases [7]. Furthermore, a number of studies have shown the prevalence of *Mycobacterium tuberculosis* with rifampicin resistance Ethiopia ranged from 4.7–18.3% [5, 8–11]. Mutations region of 81 base pairs (bp) of the rpoB gene has been found in about 96% of rifampicin (RMP) resistant *Mycobacterium tuberculosis* [8, 9].

Ethiopia is implementing a comprehensive TB/Leprosy and TB/HIV control programs and has achieved a lot in the past decade. However, In Ethiopia, the case detection rate was very low using smear microscopy in the past, but in its commitment against TB, the Ethiopian government has joined the post-2015 Global TB Strategy called “END TB strategy” which will increase case detection and further reduce the burden of this disease. To achieve these strategies Ethiopia endorsed many advanced technologies concordantly with WHO recommendations, including the implementation of the GeneXpert™ MTB/RIF assay. The assay detects *Mycobacterium tuberculosis* and rifampicin resistance by identifying mutations using three specific primers and five unique molecular probes through a rapid (2 h) process with minimal bio-safety requirements and training [12].

Ethiopia is one of the high burden countries, reflected both in its TB incidence and the estimated rates of MDR-TB [13]. However, there is limited information regarding the trend analysis of TB and rifampicin resistance in our study area. To date, there are no studies conducted that have reviewed documents systematically to identify the trends in *Mycobacterium tuberculosis* and rifampicin resistance using GeneXpert™ in Adigrat General Hospital. Therefore, this study aimed to determine the trends in *Mycobacterium tuberculosis* and rifampicin resistance using GeneXpert™ among TB-presumptive cases at Adigrat General Hospital, Eastern zone of Tigrai, north Ethiopia.

## Methods and materials

### Study design, setting, and time

A retrospective cross-sectional study design was used to collect the secondary data from June–August, 2019 at the Adigrat General Hospital. The Adigrat General Hospital is found in the Eastern zone of Tigrai, north, Ethiopia at altitude and longitude of 14°16′N 39°27′E, with an elevation of 2457 m (8061 ft) above sea level and 560 miles far from capital city Addis Ababa. In the Eastern zone based on the 2007 census conducted by central statistics agency of Ethiopia, has a total population of 755, 343, of 52.4% women and 19.34% are urban inhabitants and the majority have low income. There are 2 General Hospital, 5 primary Hospital and 37 health centers. The Adigrat General Hospital is serving as a referral for surrounding health centers and primary hospitals, and teaching center for medical and health science students. The hospital has about 120 beds and more than 250 health care providers. Adigrat General Hospital is the only hospital that testing sputum using GeneXpert*™* and treated the MDR-TB for surrounding 7 districts in the Eastern zone of Tigrai.

### Inclusion criteria/exclusion criteria

Those who had completed data in the GeneXpert*™* TB registration book were included during the study period specified and those cases with indeterminate and/or invalid results were excluded from the study.

### Dependent variable/independent variable

*Mycobacterium tuberculosis* result (Positive and Negative) and rifampicin (sensitive and resistance) were dependent variables and sex, residence, age, co-infection, site of presumptive TB, reason for diagnosis and year of diagnosis were independent variables.

### Operational definition

Presumptive-TB: An individual who presents with symptoms or signs suggestive of TB like sweating, coughing more than two weeks, loss of appetite, weight loss and weakness.

Presumptive drug resistance-TB case: refers to a person who presents with clinical features suggestive of TB or diagnosis of active TB and with either medium – or high- risk of harbor Drug resistant TB.

### Sample size

Retrospectively all presumptive TB patients from a GeneXpert*™* TB registration book from January 2015, to December 2018, were included.

### Laboratory investigation

Adigrat General Hospital TB clinic operates under the national TB- and leprosy-control program of Ethiopia, in which the diagnosis of TB is followed by GeneXpert*™* MTB/RIF assay for rifampicin resistance. Samples were processed by GeneXpert*™* MTB/ RIF (Cepheid) assay according to the manufacturer’s manual.

### Data collection and quality of data

The data were collected retrospectively from the GeneXpert*™* TB registration book in Adigart General Hospital at the Directly Observed Treatment [short course clinic] (DOTS). Data was collected using a pre-developed checklist and the quality of data was maintained by checking the completeness of necessary information; the obtained data were cross-checked and double entered and re-checked to ensure the quality of data.

### Statistical analysis and interpretation

Data obtained through the checklist and laboratory test results were double entered into the Epi-Info 3.1 software and export to SPSS*™* 20. Data analysis was performed using SPSS™ 20. Descriptive analysis, frequencies, and figures were used to explain the findings. Chi-square analysis was used to correlate categorical variables. Bivariate Logistic regression analysis was conducted primarily to check the association of each independent variable with the dependent variable at *P* value < 0.2. Multivariate logistic regression models were employed to analyze specific associations between variables. Odds ratio (OR) and 95% confidence interval (CIs) were calculated using logistic regression model to measure the strength of an association. In all cases, *p*-values less than 0.05 were considered statistically significant.

## Results

A total of 5944 presumptive TB and drug resistance TB patients was retrospectively included in this study. Among these patients, majorities were male 3455 (58.1%).The median age of the participants was 40.0 (IQR 26–57), of which the majority were in the age group 30–44 years. Of the total participants, 513 (8.6%) were HIV positive. Among the presumptive drug resistance TB patients’ majority were new case 706 (76.9%). Diagnosis of *Mycobacterium tuberculosis* using the GeneXpert™ have increased between 2015 and 2018 (Table 1).

**Table 1:** Socio-demographic and clinical characteristics of the study subjects in Adigrat General Hospital, Eastern Zone, Tigrai, North Ethiopia

Variables	Frequency	Percentage
Sex		
Female	2489	41.9
Male	3455	58.1
Residence		
Urban	5121	86.2
Rural	823	13.8
Age (Years)		
≤ 14	236	4.0
15–29	1568	26.4
30–44	1620	27.3
45–59	1107	18.6
60–74	1039	17.5
75–89	362	6.1
90	12	.2
Reason for diagnosis		
Presumptive TB	5027	84.6
Presumptive DR TB	917	15.4
Presumptive DR-TB		
New	706	76.9
Relapse	105	11.5
Failure	87	9.5
Lost to follow-up	19	2.1
Site of presumptive TB		
Pulmonary	5819	97.9
Extra-pulmonary	125	2.1
HIV status		
Negative	4761	80.1
Positive	513	8.6
Unknown	670	11.3
Year of Diagnosis		
2015	604	10.2
2016	1479	24.9
2017	1872	31.5
2018	1989	33.5

The overall positivity of *Mycobacterium tuberculosis* among all forms of presumptive TB patient was 24.3% (1446/5944). The *Mycobacterium tuberculosis* positivity rate was highly observed in the age group 30–44 years, with 420 cases (26%). Twenty three percent (1188/5027) and 28% (258/917) presumptive of TB and DR-TB, respectively, were diagnosed with *Mycobacterium tuberculosis*. MTB/HIV co-infection was observed in 33.3% (171/513) of the involved patients. The trends of the positivity of *Mycobacterium tuberculosis* almost similar in the first three years but were considerably higher by 2018. Correlation analysis of MTB showed a strong association with age, the reason for diagnosis, site of sample collection and being HIV infected (Table 2).

**Table 2:** Trends of positive M*. tuberculosis* among presumptive TB patients diagnosed in Adigrat General Hospital using GeneXpert™ MTB/RIF assay

Variables	*M. tuberculosis result by GeneXpert™*		Total	AOR (95% CI)	*P*-value
	*Detected (%)*	*Not-Detected (%)*			
Sex					
Female	596 (24.0)	1893 (76.0)	2489 (41.9)	a	
Male	850 (24.6)	2605 (75.4)	3455 (58.1)	0.99 (0.88–1.12)	0.86
Residence					
Urban	1266 (24.7)	3855 (75.3)	5121 (86.2)	a	
Rural	180 (21.9)	643 (78.1)	823 (13.8)	1.08 (0.90–1.29)	0.41
Age (Years)					
≤ 14	44 (18.6)	192 (81.4)	236 (3.9)	2.38 (0.299–18.95)	0.41
15–29	393 (25.1)	1175 (74.9)	1568 (26.4)	3.26 (0.42–25.29)	0.26
30–44	420 (26.0)	1200 (74.0)	1620 (27.3)	3.66 (0.47–28.39)	0.22
45–59	258 (23.3)	849 (76.7)	1107 (18.6)	3.81 (0.49–29.67)	0.20
60–74	235 (22.6)	804 (77.4)	1039 (17.4)	3.64 (0.47–28.32)	0.22
75–89	93 (25.7)	269 (74.3)	362 (6.1)	4.08 (0.52–32.05)	0.18
90	3 (25.0)	9 (75.0)	12 (0.3)	a	
Reason for diagnosis					
Presumptive TB	1188 (23.6)	3839 (76.4)	5027 (84.6)	a	
Presumptive DR TB	258 (28.1)	659 (71.9)	917 (15.4)	1.63 (1.40–1.90)	< 0.01^b^
Presumptive DR-TB					
New	1385 (23.3)	4348 (73.1)	5733 (96.4)	a	
Relapse	31 (29.5)	74 (70.5)	105 (1.8)	2.69 (0.73–10.04)	0.14
Failure	27 (31.0)	60 (69.0)	87 (1.4)	1.68 (0.489–5.80)	0.41
Lost to follow- up	3 (15.8)	16 (84.2)	19 (0.4)	2.79 (0.76–10.23)	0.12
Site of presumptive TB					
Pulmonary	1414 (24.3)	4405 (75.7)	5819 (97.9)	a	
Extra-pulmonary	32 (25.6)	92 (74.4)	125 (2.1)	0.70 (0.48–1.03)	0.70
HIV status					
Negative	1128 (23.7)	3633 (76.3)	4761 (80.0)	a	
Positive	171 (33.3)	342 (66.7)	513 (8.7)	1.67 (1.35–2.07)	< 0.01^b^
Unknown	147 (21.9)	523 (78.1)	670 (11.3)	0.97 (0.77–1.21)	0.77
Year of Diagnosis					
2015	142 (23.5)	462 (76.5)	604 (10.2)	0.75 (0.61–0.93)	0.08
2016	339 (22.9)	1140 (77.1)	1479 (24.9)	0.63 (0.54–0.74)	< 0.01
2017	425 (22.7)	1447 (77.3)	1872 (31.5)	0.65 (0.56–0.75)	< 0.01
2018	540 (27.1)	1449 (72.9)	1989 (33.4)	a	

^*a*^*-Reference category M. tuberculosis- Mycobacterium tuberculosis, DR TB -drug resistant tuberculosis and AOR-Adjusted odd ratio*, ^b^*Significantly associated*

From the total confirmed of all forms of presumptive TB cases, 9.1% (132/1446) were resistant to rifampicin, of which 8.7% (103/1188) and 11.2% (29/258) where presumptive TB and presumptive drug resistance-TB respectively. Of the total confirmed positive cases 8.7% (103/1385) and 11.2% (29/258) were rifampicin resistance of presumptive tuberculosis and presumptive drug resistance tuberculosis patients respectively. Of the total TB-HIV co-infected patients, 15.2% (26/171) where rifampicin resistance. The trends of rifampicin-Resistant were seen as a minimum variation from year to year, with the minimum observed in 2018 and the maximum in 2015. The sensitivity and resistance of rifampicin results showed a statistically significant difference with reason of diagnosis and HIV status (Table 3).

**Table 3:** Multivariable analysis of rifampicin-resistant among the total *Mycobacterium tuberculosis* cases using GeneXpert™ MTB/RIF assay, in Adigrat General Hospital

Variables	*Pattern of RIF*		Total N (%)	AOR (95% CI)	*P*-value
	*Sensitive N (%)*	*Resistant N (%)*			
Sex					
Female	535 (89.8)	61 (10.2)	596 (41.2)	a	
Male	779 (91.6)	71 (8.4)	850 (58.8)	0.74 (0.5–1.09)	0.13
Residence					
Urban	1151 (90.9)	115 (9.1)	1266 (87.5)	a	
Rural	163 (90.6)	17 (9.4)	180 (12.5)	1.10 (0.61–1.99)	0.74
Age (Years)					
≤ 14	41 (93.2)	3 (6.8)	44 (3.0)	a	
15–29	376 (95.7)	17 (4.3)	393 (27.2)	1.12 (0.37–3.36)	0.84
30–44	386 (91.9)	34 (8.1)	420 (29.1)	0.52 (0.16–1.53)	0.23
45–59	225 (87.2)	33 (12.8)	258 (17.8)	0.54 (0.18–1.62)	0.27
60–74	205 (87.2)	30 (12.8)	235 (16.3)	0.53 (0.17–1.60)	0.26
75–89	78 (83.9)	15 (16.1)	93 (6.4)	1.48 (0.46–4.74)	0.51
≥ 90	3 (100)	0	3 (0.2)		
Reason for diagnosis					
Presumptive TB	1085 (91.3)	103 (8.7)	1188 (82.1)	a	
Presumptive DR TB	229 (88.8)	29 (11.2)	258 (17.9)	8.92 (5.81–13.69)	< 0.01^b^
Presumptive DR-TB					
New	1256 (90.7)	129 (9.3)	1385 (95.7)	a	
Relapse	30 (96.8)	1 (3.2)	31 (2.2)	0.32 (0.02–4.71)	0.41
Failure	26 (96.3)	1 (3.7)	27 (1.9)	0.16 (0.01–1.85)	0.14
Lost to follow-up	2 (66.7)	1 (33.3)	3 (0.2)	0.16 (0.01–2.49)	0.19
Site of presumptive TB					
Pulmonary	1283 (90.7)	131 (9.3)	1414 (97.8)	a	
Extra-pulmonary	31 (96.8)	1 (3.2)	32 (2.2)	0.62 (0.22–1.76)	0.37
HIV status					
Negative	1036 (91.8)	92 (8.2)	1128 (78.1)	a	.
Positive	145 (84.8)	26 (15.2)	171 (11.8)	1.97 (1.13–3.44)	0.02^b^
Unknown	133 (90.5)	14 (9.5)	147 (10.1)	0.88 (0.45–1.72)	0.72
Year of Diagnosis					
2015	123 (86.7)	19 (13.4)	142 (9.8)	2.24 (1.19–4.24)	0.01
2016	310 (91.4)	29 (8.6)	339 (23.5)	0.91 (0.53–1.55)	0.72
2017	386 (90.8)	39 (9.2)	425 (29.4)	1.20 (0.74–1.96)	0.47
2018	495 (91.7)	45 (8.3)	540 (37.3)	a	

^*a*^*-Reference category, RIF -rifampicin resistant, DR TB -drug resistant tuberculosis, M. tuberculosis- Mycobacterium tuberculosis and AOR-Adjusted odd ratio,* ^b^*Significantly associated*

**Fig. 1:**
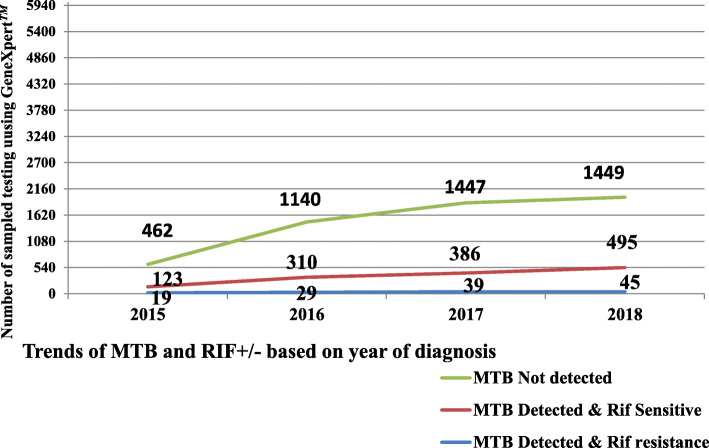
Trends of *Mycobacterium tuberculosis* and rifampicin resistant in Adigrat General Hospital, eastern zone of Tigrai, Northern Ethiopia

The trends of positivity in *Mycobacterium tuberculosis* and rifampicin resistance were minimum variation between 2015 and 2018. In 2015, *Mycobacterium tuberculosis* were found in 142/604 (23.5%) of whom 19/142 (13.4%) were rifampicin-resistant, but by 2018 *Mycobacterium tuberculosis* incidence was 540/1989 (27.1%) of whom 45/540 (8.3%) were rifampicin-resistant. In general, rifampicin-resistant in January 2015, 2016, 2017 and till December 31, 2018 were shown 13.4, 8.6, 9.2 and 8.3% respectively Fig. 1.

## Discussion

The WHO continues to search for innovative technologies to enhance accurate and reliable laboratory diagnosis of TB to curb *Mycobacterium tuberculosis* and DR-TB worldwide. However, the emergence of drug-resistant forms of TB, which need more resources to detect, treat, and effectively reduce the burden of disease is a challenging problem. GeneXpert™ MTB/RIF assay is a new automated real-time Nucleic Acid Amplification Technology that overcomes many of the current operational difficulties in TB diagnosis [14].

TB affects mostly adults in the economically productive age groups with approximately two-thirds of cases estimated to occur among people aged 15–59 years [1].

In the present study, the overall forms of presumptive *Mycobacterium tuberculosis* positivity rate were 24.3%. Our finding was similar to studies conducted in the Debre Markos Hospital (23.2%) [15], Gondar Referral Hospital (24.6%) [16], Gambella (20.0%) [17], Afar (24.5%) [18], India (27.6%) [4], South Africa (26%) [19], Nigeria (22.9%) [20] And the WHO report in Africa (25%) [2]. However, it was lower compared to reports in Jigjiga (65.5%) [21], Kenya (32.25%) [22], in eastern Uttar Pradesh (32.9%) [23] and Congo (79.1%) [24]. The main difference in these latter findings may show our inclusion of all forms of presumptive tuberculosis while other studies included identified cases of *Mycobacterium tuberculosis*. In contrast, our finding was higher when compared with studies conducted in Metema and Armacho (5.7%) [7], Felege Hiwot Referral Hospital, and Debre Tabor Hospital (14.6%) [25], in three referral hospitals and the regional laboratory in Addis Ababa (15.11%) [26], other parts of Ethiopia (4.7–10.8%) [27–29], Nigeria (10.3%) [30] and India (2.31%) [31]. The variations might be due to the difference in study design, type and number of participants, and environmental conditions.

The co-infection of TB-HIV in this study was found to be high at 33.3% (171/513). This finding was supported by previous studies conducted in Amhara (27.7%) [32], Gambella [17], in Ethiopia (29.4%) [33], and in Central Nigeria (36.3%) [34]. However, our findings were higher than studies conducted in the Debre Markos Referral Hospital (16.6%) [15], different studies across Ethiopia (20.3–24.2%) [16, 35–38], and a WHO estimation for Ethiopia of 14% (9.6–19%) [2]. Conversely, the findings were lower than studies conducted in the Felege Hiwot Referral Hospital and Debre Tabor Hospital (41.9%) [25], Zambia (98.3%) [39], and South Africa (> 70%) [40]. The possible explanations for this difference could be reflect policy recommendations for which HIV infected patients, as an eligible group, are more likely to be tested using GeneXpert™.

In this study, *Mycobacterium tuberculosis* was prevalent in all ages, but have seriously hit the age group of 30–44 years with 26.0% and of whom 34/420 (8.1%) were rifampicin resistant. The positivity finding was in line with studies conducted in Gondar (29.8%) [16], different studies in Ethiopia [27–29, 41], WHO reports 2017 [2] and Agaro Teaching Health Center in southwestern Ethiopia [42]. However, contrary findings with several studies in a different part of Nigeria and Zambia [22, 30, 39] which had lower prevalence, but higher than a study conducted in eastern Uttar Pradesh (40%) [23].

In the present study, the percentage of *Mycobacterium tuberculosis* positivity significantly higher in presumptive TB patients (20.0%) compared to presumptive drug resistance (4.3%) with (*P* < 0.00). This finding was comparable to studies conducted in Afar (20.9) [18], Debre Markos Referral Hospital (15.1%) [15] and Gambella (19.6%) [17]. However, it is much lower than studies conducted in Felege Hiwot Referral Hospital and Debre Tabor Hospital (54.8) [25], Gondar (25.2%) [16] and Zimbabwe (37.1%) [43]. The discrepancies might be due to our inclusion of all presumptive TB cases, and a high number of participants.

According to our study, we found 132 (9.1%) of rifampicin-resistant cases among confirmed TB cases. This result is comparable with studies conducted in Debre Markos Referral Hospital (10.3%) [15], Felege Hiwot Referral Hospital and Debre Tabor Hospital (9.3%) [25], Addis Ababa (9.9%) [26] and India (10.5%) [44]. Our finds are higher than studies conducted on northwest, east and south parts of Ethiopia (2.9–5.7%) [7, 17, 23, 26, 37, 38, 45, 46], Nigeria (2.9%) [30] and Zambia (5.9%) [39]. The possible explanation for these variations could be related to our retrospective approach spanning four years, or differences in study designs. However, our incidence was lower than for studies conducted in Gondar 15.8% [16],other parts of Ethiopia (11.5–39.4) [45, 47, 48],Congo (42.2%) [24] and China (17.6–26.3%) [6, 49].

### Limitation of the study

As we collected retrospective data from the GeneXpert*™* TB registration book, we encountered data missing and incompleteness. Variables included for associated factors were also limited.

## Conclusion

In our study, the overall trends of *Mycobacterium tuberculosis* and rifampicin resistance were found to be high and with minimum variation each year. Rifampicin resistance is more common in patients with HIV and presumptive drug resistance tuberculosis individuals. Therefore, maximizing early detection of drug-resistant and strengthening tuberculosis infection control activities are recommended to reduce the burden of this contagious and potentially deadly disease.

## Data Availability

The findings of this study are generated from the data collected and analyzed based on the stated methods and materials. All the data are found in the manuscript and there are no supplementary files. The original data supporting this finding will be available upon request through the corresponding author.

## Abbreviations

ADU: Adigrat University
AGHL: Adigrat General Hospital Laboratory
DNA: Deoxyribonucleic Acid
DOTS: Directly Observed Treatment, short course
HIV: Human Immunodeficiency Virus
M. tuberculosis: *Mycobacterium tuberculosis*
MDR-TB: Multidrug Resistance tuberculosis
RIF: Rifampicin
RNA: Ribonucleic Acid
TB: tuberculosis
WHO: World Health Organization
XDR TB: Extensively drug resistant tuberculosis
